# Bedrock: Reading the Death Drive in the Time of the Anthropocene

**DOI:** 10.17613/wbfp0-gjt79

**Published:** 2025-02-01

**Authors:** Laura Salisbury

**Affiliations:** 1 University of Exeter, U.K.

**Keywords:** Freud, death drive, time, Anthropocene, geological

## Abstract

Sigmund Freud’s death drive tends to be invoked in ecocritical thought as a bedrock truth that explains how the drive for pleasure, mastery, and expansion within and for the human organism is inherently bound to aggression and destructiveness. But a close reading of Freud and the deviations and repetitions of drive suggests an ongoing relationship and collective alliance between life and death rather than any simple victory for destruction. This article argues that attending to the more-than-human form and force that underpins yet simultaneously resists all human projects – what Freud describes as the unanalysable “bedrock” of the death drive – offers ways of recasting the temporalities of Anthropocene imaginaries. Using the work of Catherine Malabou, Elizabeth Povinelli, Lisa Baraitser, and Édouard Glissant, this article rethinks the resistant bedrock of the death drive and its complex interweaving of life with nonlife, as a way of decentering and “de-dramatizing” formations of the human. The article suggests that attending to how things come to matter over time opens up the possibility of suspending forms of action that mortgage the existence of many human and more-than-human others to the sustaining of a certain configuration of Life only, to produce instead a time where things might be able to die in their own fashion.

In a recent essay, Anna Kornbluh has highlighted a turn in cultural theories of climate catastrophe and ecocide toward what gets represented as a “bedrock psychic truth” (2024, para. 13). There is a repetitive engagement, even if sometimes just a glancing one, with one of Sigmund Freud’s most elusive concepts. This concept seeks to explain how the drive for pleasure, mastery, and expansion within and for the human organism may also be inherently bound to aggression and destructiveness. In other words, there is a turn to the death drive. Kornbluh suggests that the death drive offers an all too tempting explanation of “the unholy convergence of best-practice aggression and the growth imperative of extractive capitalism” (para. 4) that produces ecological destruction. “[I]t is our nature to annihilate nature” (Kornbluh, para. 5); or so the argument goes. However, Kornbluh resolutely refuses the idea of the death drive as an unanalysable bedrock truth of climate catastrophe. She points out, first, and following many others, that not all humans are equally responsible, either historically or currently, for the levels of carbon emissions linked to climate catastrophe.^2^ She then goes on to argue that the death drive is much more dynamic than many of those who invoke it imply. Inextricably bound to the life instincts, the death drive is subject to a movement that produces multiple and paradoxically creative new relations rather than an inanimate achievement or something that is simply a thing in itself and nothing else. Drive, she argues, never simply is what it is. Freud himself asserts that the story of human civilization and its inevitable discontents is a relational one: it is the “struggle between Eros and Death, between the instinct of life and the instinct of destruction, as it works itself out in the human species” (Freud, 1961b, p. 122). And for Kornbluh, following Freud, the deviations and repetitions of drive, understood as sites of struggle that play out over time, ensure an ongoing relationship between life and death from which something other than simply catastrophe might unfold. For drive, understood as naming a set of relations rather than destinations, can also be aligned with what delays or impedes the achievement of a quick death. As Kornbluh argues, the death drive ensures that there are also forms of “mitigation” that “[keep] life keeping on, in new cul-de-sacs, through secondary satisfactions, institutional workarounds, and aleatory indirections. It is too late, but things can still be less worse” (para. 22). There are creative alliances being made, and to be made, with the death drive.

The question of time haunts Kornbluh’s essay. It is too late: death is always already the endpoint, both for individual organisms and whole ecosystems. Various tipping points for species survival have been reached and breached. Nevertheless, life, even if not in all its forms, goes on. Perhaps, then, there remains something still to be done amidst the nothing to be done, especially, Kornbluh suggests, if one thinks with the death drive and its temporalities rather than accepting them as versions of destructive fate or ends already timelessly inscribed into the beginning. This article thinks through how tarrying with the more-than-human form and force that underpins yet simultaneously resists all human projects – what Freud describes as the “bedrock” of the death drive – might offer ways of recasting Anthropocene imaginaries that bind together seemingly incommensurable temporal scales: the geological that exceeds human individual and even species timeframes, and the historical scene of action and human responsibility (Chakrabarty, 2009).^3^ For the concept of the Anthropocene, like the death drive’s invocation of biological prehistory, works to suture an enduring trace and expression of deep time to the actions and experiences of historical time, including the time of individual lives. This article seeks to open up what it means to ground destructiveness in forms and temporalities that exceed human timescales, but that nevertheless remain woven together with the ongoing time of human lives. It also hopes to preserve the complexity of the ground that is being invoked in a grounding gesture, to track the possibilities of making a creative alliance with the death drive’s repetitions, inertia, temporal relationality, and even bedrock opacity. These creative alliances demand remaining cautious of any attempt to bind ecological ethics to the putative biological facticity of drive: an assertion that it is simply our nature either to destroy or even to preserve nature. But they might make something of Leo Bersani’s cryptic, elliptical suggestion that sustaining awareness of a relationship with the death drive might underpin an “ecological ethics” in which “the subject, having willed its own lessness, can live less invasively in the world” (Bersani, 2002, p. 30).^4^ There might be relationships both with and within the death drive that could run counter to mastery, expansion, and extraction.

## Grounding

Returning to Freud looks suspiciously like a grounding gesture, but Freud on the death drive does not exactly yield *terra firma*. In 1920, Freud’s *Beyond the Pleasure Principle* introduced the concept of the death drive which, “though it does not contradict the pleasure principle, is nevertheless independent of it and seems to be more primitive” (Freud, 1955, p. 32). Freud finds in the compulsion to repeat a fundamental urge “*to restore an earlier state of things*,” which is understood as “the expression of the inertia inherent in organic life” (p. 36). The desire to return to “the quiescence of the inorganic world” (p. 62) takes Freud back to the most primordial articulations of life, even as he also sees it at work within world history and the individual suffering of traumatized soldiers in World War I. But the life instincts of mastery and self-preservation that undergird the pleasure principle do not simply work in opposition to death; instead, Freud comes to insist that “*the aim of all life is death*” (p. 38). The self-preservative instincts do not simply contradict the death drive; rather, the life instincts form a relation and alliance with it:

They are component instincts whose function it is to assure that the organism shall follow its own path to death, and to ward off any possible ways of returning to inorganic existence other than those which are immanent in the organism itself […] What we are left with is the fact that the organism wishes to die only in its own fashion. Thus these guardians of life, too, were originally the myrmidons of death. Hence arises the paradoxical situation that the living organism struggles most energetically against events (dangers, in fact) which might help it to attain life’s aim rapidly – by a kind of short-circuit. (Freud, p. 39)

Instead of one group of instincts achieving victory over the other, life, as it expresses itself over time, requires remaining in a vital, paradoxically creative relation to the death drive, represented here as the underlying constant of psychic life. As a consequence:

It is as though the life of the organism moved with a vacillating rhythm. One group of instincts rushes forward so as to reach the final aim of life as swiftly as possible; but when a particular stage in the advance has been reached, the other group jerks back to a certain point to make a fresh start and so prolong the journey. (Freud, p. 40-41)

The journey of living is extended because life and death remain fundamentally bound to each other. According to its drive toward death, the organism exhausts itself as an expression of the continuation of life.

The suggestion that satisfaction comes not from achieving aims straightforwardly but from diverting them shapes the death drive into something that unravels both humanity’s sense of itself and Freud’s own hopes for psychoanalysis as a curative clinical practice. As Todd McGowan argues, the Freud of *Civilization and Its Discontents* (1930), who is facing up to the death drive, comes to understand a straightforwardly “utopian future [to be] unimaginable” (2024, p. 99). By the end of “Analysis Terminable and Interminable” (1937), Freud suggests, in what is close to a last word, that there are certain bedrocks upon which psyches stand and on which analysis founders:

We often have the impression that with the wish for a penis and the masculine protest we have penetrated through all the psychological strata and have reached bedrock [*Felsens*], and that thus our activities are at an end. This is probably true, since, for the psychical field, the biological field does in fact play the part of the underlying bedrock. The repudiation of femininity can be nothing else than a biological fact, a part of the great riddle of sex. It would be hard to say whether and when we have succeeded in mastering this factor in an analytic treatment. We can only console ourselves with the certainty that we have given the person analysed every possible encouragement to re-examine and alter his attitude to it. (Freud, 1964, pp. 252-53)

One “field” on which psychoanalysis breaks apart as a method is the “biological fact” of the repudiation of femininity, figured as passivity, in both men and women. The other psychic bedrock is the death drive, which is both the expression of species-level aggressive instincts, a desire for mastery, and part of the repetitive return to sites of psychic dissolution or unbinding, or what Lisa Baraitser names the “ultimate passive state” (2020, p. 507; 2023, p. 913-914).

The ambivalence of Freud’s late writing obtrudes in a resistant fashion, with arguments stumbling, deviating, and repeating. Although the repudiation of femininity and the death drive may be the bedrock upon which clinical analysis dashes itself, the relationship of analytic practice to a patient’s unanalysable material simultaneously produces repetition and interminability, pushing analysis, like life, to exhaust itself “in its own fashion” (Freud, 1955, p. 39). The compulsion to repeat does not simply mark the death of analysis, either for an individual or as a practice; instead, it suggests the possibility of an oddly creative interminability. As Adrian Johnston (2005) has shown in detail, despite Freud’s insistence on the timelessness of the unconscious across much of his work,^5^ the metapsychological concept of drive needs to be understood as internally bifurcated and differential – as a matter of time. Committed both to repetition and to deviation and development, Johnston indeed describes how drive is “split along the lines of two irreconcilable, incompatible axes – an *axis of iteration* (source-pressure) and an *axis of alteration* (aim-object)” (p. 149). As Johnston argues, these axes simultaneously guarantee and confound one other: “The axis of iteration’s demand for a pure repetition unaffected by temporal mediation can never be satisfied, since it necessarily passes through the matrix of the axis of iteration’s temporal processes” (p. 150). Absolute repetition is impossible because of the temporal difference through which repetition is underwritten; alteration appears because absolute repetition is impossible for an organism exposed to the reality of experiences over time marked and structured by difference. It is precisely this temporal potential that opens the death drive’s workings not to a utopian future, exactly, but to what Kornbluh describes as diverse mitigations where things might be “less worse,” or what Todd McGowan (2024) names a disruptive “space,” emergent over time, that enables ethical action precisely because it suspends what appears as immediate self-interest. Is it possible, then, to imagine an alliance with the death drive that would enable an interruption of the version of the human that has cast itself as a geological agent capable of marking the bedrock of the planet?

## Plasticity and Lithicity

Freud never gave up on analysis; but he also never quite gave up on the idea that a bedrock that undergirds psychic life, civilizations, and of course their discontents, might one day be mappable via neurobiology. Throughout his career, Freud suggested that psychoanalysis might only be a staging post on the journey to an empirically tractable and neurologically grounded account of mind. In 1914, he wrote that “[w]e must recollect that our provisional ideas in psychology will presumably some day be based on an organic substructure” (Freud, 1957, p. 78). Even in the metapsychological *Beyond the Pleasure Principle*, he asserted that “[b]iology is truly a land of unlimited possibilities. We may expect it to give us the most surprising information and we cannot guess what answers it will return in a few dozen years to questions we have put to it” (Freud, 1956, p. 60). As we have seen, Freud notes that “for the psychical field, the biological field does in fact play the part of the underlying bedrock” (1964, p. 252). Neurobiology will one day have become an “organic substructure” that truly grounds the relationship between the organic and the inorganic that constitutes the workings of the death drive.

Just as the concept of the Anthropocene has a force because it marks anthropogenic effects on the climate in the bedrock – in the empirically observable world that is usually taken to be impervious to human action (Crutzen and Stoermer 2000) – an evolved neurobiological substrate suggests a facticity that stabilizes and outlasts the cultural variegations and individual plasticity of the psyche. In 2017, the philosopher Catherine Malabou responded to an invitation to work explicitly with this point of contact, placing her philosophical use of neurobiological substrates alongside the concept of the Anthropocene. In “The Brain of History, or, The Mentality of the Anthropocene,” she sets out the problem, following Chakrabarty’s influential analysis (2009):

The Anthropocene situates the human being itself between nature and history. On the one hand, it is still of course the subject of its own history, responsible, and conscious. Consciousness of history, or “historicity,” is not separable from history itself. It entails memory, capacity to change, and, precisely, responsibility. On the other hand, though, the human of the Anthropocene, defined as a geological force, must be seen as neutral and indifferent, as a geological reality itself. The two sides of this new identity cannot mirror each other, causing a break in reflexivity. (Malabou, 2017, p. 40)

The Anthropocene introduces a split between the conscious, responsible human subject of history, and a “blind” geological force that is radically asubjective and materially traversed by forces and scales that cannot be experienced by the senses or assimilated into human narratives of continuity or responsibility.

Malabou proposes, however, that the underlying neurobiology of the human might bridge this break. She suggests the possibility of reckoning with the temporality of the Anthropocene through her sense of the human brain as an “essential intermediary between the historical, the biological, and the geological” (Malabou, 2017, p. 45) – something that mediates the bidirectionality of influences between the genetic, environmental, subjective, and socio-cultural. Malabou insists that although the brain develops via slow evolutionary adaptations, it is also embedded within a socio-cultural environment that produces epigenetic change within historical time. For example, the emergence of forms of agriculture that render more calories available for consumption alters brain-body states, producing new needs, addictions, and desires that cut across biological and cultural boundaries, and new social structures that further environmental change. Malabou thus argues for the material “embedment” of the human brain within a diversity of non-linear feedback loops with the environment. “Embedment” is Lambros Malafouris’s term that “derives from the fusion of the terms ‘embodiment’ – referring to the intrinsic relationship between brain and body— and ‘embeddedness’ – describing the intrinsic relationship between brain/body and environment” (2010, p. 52).^6^ In this model, there is an insistence on multiple forms of relationality, and the emergence of neuronal matter over and across various temporal scales. Malabou thus invokes a renewed concept of “mentality,” located “intermediarily” between the inorganic and the neural:

The history of mentality also includes, as one of essential dimension, the materiality of inorganic nature, the soil, the rocks, the mountains, the rivers, the earth. A mentality is a hybrid concept that comprehends not only the psychic and the social but also the originary likeliness of the mind and the fossil, the inscription of naturality in thought and behavior. Mentality, in that sense, is rooted in the brain and not in consciousness. (Malabou, p. 51)

The human brain needs to be understood as socially, psychically, biologically, and environmentally formed through temporalities and processes that necessarily exceed what is conventionally figured as the territory of the human, even as they are nevertheless also experienced within individual lives and historical time. This mentality that emerges in the suspension of consciousness needs to form itself into new configurations that can make something of its embedment within the slow, inert, and resistant temporality of the geological, rather than simply remaining addicted to its own numbness and indifference, and to temporalities that exceed the time frames of historical responsibility.

Malabou suspends the philosophical move that would turn her reflections on a “neutralized,” “geologized” consciousness (2017, p. 48) into a programme of responsible action that could mitigate ecocide and the climate catastrophe the concept of the Anthropocene is designed to invoke. But she does suggest some openings when she writes that “addictive processes have in large part caused the Anthropocene, and only new addictions will be able to partly counter them” (Malabou, p. 47). The essay implies that humans might come to understand themselves as geological agents that nevertheless inhabit historical time through what she marks as the essential plasticity of the human brain – its capacity to change and hold form and to destroy previous ways of being (Malabou, 2008; Malabou, 2009) – that might then enact new relationships, new feedback loops, new addictions, and new expressions of embedment. Although Malabou’s essay is in many ways more theoretically aligned with her work on epigenesis than her extensive body of work on neurological plasticity, her determination that “only the setting of new addictions can help in breaking old ones” (2017, p. 49) suggests that further attention might usefully be paid to her theory of the brain’s destructive plasticity – what she describes elsewhere as a neuronal death drive.

Malabou’s concept of destructive neural plasticity depends on a recasting of the Freud’s death drive in more radically material terms. In The New Wounded (2012), she finds in the revolution within brain and psyche that appears following neurological damage, something that fundamentally threatens the notion of a more-or-less continuous, autobiographical subject that can be psychoanalysed according to its configurations of repetition and deviation. This version of neurological rather than psychological trauma, Malabou argues, figures the ever-present possibility of a violent interruption by something completely unexpected that cannot be reconciled with a pre-existing sense of selfhood and psychic continuity. She argues this against Freud, for whom the brutal unexpected encounters with the external world owe their traumatic impact to the way they touch a psychic reality already established in the past and shaped according to the primacy of sexuality.

*Contra* Freud, Malabou suggests that brain damage is a form of wounding, of trauma, from which no previous narrative or immanent psychic meaning can be reconstructed. The material neurological substrate can be fundamentally damaged by head wounds, non-traumatic injuries with an internal source (e.g., stroke) or external source (e.g., infection), or neurodegenerative conditions like dementia: all have the potential to alter irrevocably the appearance and sensation of a selfhood structured by past experiences. These new wounds, as Malabou terms them, cannot be made to resonate with a psychical reality that precedes or potentially outlasts them. The ground is radically reformed and there is no going back. As Malabou argues:

Whereas the psychoanalytic narrative of the resistance of the indestructible within destruction privileges the persistence of childhood, the past, and psychic destiny, which become recognizable through the disturbance that solicits them, the neurological narrative of the destruction of the resistance to destruction stages the exhausted but surviving resources of a psyche that no longer recognizes itself. (2012, p. 56)

As Adrian Johnston and Malabou restate it: “Neurobiology puts this so-called psychic immortality into question […] the primitive psyche is not imperishable, as Freud states; it can be damaged without any return to a previous state” (2013, p. 61). Because of this fundamental interruption, Malabou implies that these traumas have little in common with the dream (*Traum*), always available for analysis. Instead, brain trauma like a cerebral lesion cannot “speak”; it is a wound without a meaningful past and without what she would frame as a hermeneutic future. It implicates inorganic numbness and indifference within the structure of brain and its functioning, forming an inert break within the psyche’s conscious and affective life.

Malabou critiques Freud for his “*failure to admit the existence of a beyond of the pleasure principle*” (2012, p. 189), or, in other words, his failure to face a truly radical encounter with the death drive. Freud’s “beyond” is unprepared to accede to the force of wounds that do not achieve meaning by resonating with an already present, indestructible internal world. Malabou thus determines that the subject needs to be reconceived as holding within its material nature the capacity for a creative self-destruction, for an eruption that breaks from the consciousness of the past, and for a reforming of experience in relation to a radical externality that interrupts the narratives of autobiographical continuity underpinning current ideas of responsibility, action, and change.

Reading “The Brain of History, or, The Mentality of the Anthropocene” alongside Malabou’s earlier work on destructive neural plasticity shapes the ground, albeit tentatively, for one of the few imperatives in the essay: that “[e]cology has to become a new libidinal economy” (2017, p. 49). Although Malabou does not outline what this might look like, we can infer that new relationships, and new alliances, need to be formed with drive and its addictions, that can use the “the originary likeliness of the mind and the fossil” (p. 51), or the embedment of mind and brain within the movements and frames of deep geological and evolutionary time,^7^ to shape new ways of being in historical time. For Malabou, these new ways of being would, crucially, still be part of the nature, or the biological facticity, of the human. And as Cate Reilly (2025) has shown, the account of brain matter and functioning within each of Malabou’s theories is dehistoricized precisely because its status as a biological fact is crucial to the argument. Nevertheless, in her return to a bedrock of embedment, Malabou implies a biologically underpinned theory of drive that suggests a capacity to break from the foregrounding of the actions and relational structures that have grounded certain version of the Eurocentric, Enlightenment human subject. Even though she binds the concept of embedment and the capacity for radical change into the biological nature of the human, Malabou animates, or renders plastic, the facticity of a neuronal substrate with feedback loops, temporality, relationality, and with the destructive plasticity that suggests a capacity for radically other ways of being.

Animating the temporal sequencing of the bedrock pushes in a suggestive way against the stratigraphic imaginary that Kathryn Yusoff rightly detects in Freud’s work (2024, p. 248-49). Yusoff defines stratigraphy as:

the study of layered materials (strata) that were deposited over time – their lateral and vertical relations, as well as their composition. The basic law of stratigraphy, the law of superposition, states that lower layers are older than upper layers, unless the sequence has been disturbed. (2024, p. 242)

We can note that in using strata as a fundamental grounding metaphor in psychoanalysis, Freud stayed true to his neurological training. He critiqued but nevertheless built on localizationist neurological models in his pre-psychoanalytic writings that framed the brain as territory to be discovered and mapped. Freud was also significantly influenced by the neurologist John Hughlings Jackson (Greenberg, 1997), who proposed the existence of different levels of neurological functioning, with expression by older and less conscious levels inhibited by younger controlling mechanisms higher up the nervous system. In this model, brain and psyche were formed, as a “biological fact,” into “primitive” strata covered by the newer, more evolutionarily developed, “higher” parts of mental and neurological matter and functioning.

Yusoff draws our attention to how psychoanalysis, alongside many other systems of knowledge in the period, became bound into a racialized, colonial imaginary. Flows are compressed and solidified into strata; the project of nineteenth-century colonial Europe, with its geological imaginaries of resource extraction and hierarchies of value, become mapped on to brain and mind. This is rendered all too clear in one of Freud’s notorious accounts of the bedrock of feminine passivity. In *The Question of Lay Analysis*, Freud wrote: “We know less about the sexual life of little girls than of boys. But we need not feel ashamed of this distinction; after all, the sexual life of adult women is a ‘dark continent’ for psychology” (1959, p. 212). Borrowing the idea of Africa as hostile, impenetrable, and far from the light of rationality from colonial explorer John Rowlands Stanley’s description of Africa as The Dark Continent (1878), Freud shapes women’s sexual desires into an enigma associated with, amongst other things, the imagined “passivity” and “primitive” state of the uncolonized world (Baraitser, 2023, p. 915). Whether a virgin forest or a bedrock, these unconscious, unknowable elements are transformed into a resource from which conscious work on the surface will extract value, although, as we have seen, they also represent the site where the mission of scientific understanding risks breaking apart. Freudian psychoanalysis makes much of the metaphor of archaeology, as excavation brings hidden objects to life and “*[s]axa loquuntur! [stones speak!]*” (Freud, 1962a, p. 192); but the feminized and racialized geological bedrock, uncovered but unworked by human hand, signals something that resists hermeneutics and the twists and turns of representation upon which psychoanalysis is built. Some stones, like some wounds, do not easily speak.

Freud’s use of the metaphor of bedrock forms part of the longstanding use of stone as a conventional and unexamined metaphor for facticity: nature’s rebuke to the human attempt to form it after its own mental shapes. Stone can all too easily become, in Jeffrey Cohen’s words, “a symbol for that which is bluntly real, a synonym for mere thingness, a figure for recalcitrance, even silence” (2015, p. 16). But when observed from the non-anthropocentric perspective the lithic has the capacity to induce (Edwards, 2023), stone and rock disclose a more-than-human liveliness through the animation of multiple and contradictory temporalities: the “slow sedimentation of alluvium and volcanic ash, grinding tectonic shift, crushing mass and epochal compaction, infernal heat, [and the] relentless turbidity of the sea” (Cohen, p. 4). As Cohen argues, rock has a particular form of “density, extensiveness, tempo, and force [that makes it] actively unknowable” (p. 8); but it shudders with movements, shapes, and temporalities that have persistently made alliances with the liveliness of human projects and imaginaries. Much of the calcium carbonate of the marble used in statuary was once living – the shell and bone of sedimentary limestone – before it re-crystallized under heat and pressure. In his account of the power of mineral within the imagination, Gaston Bachelard also suggests that salt, as Paracelsus’s third universal principle, is not “mere thingness”; rather, it “symbolizes the ties that constitute a *body* of any kind, stone or human. Salt is valued as a principle of active *concentration*” (2002, p. 203). Rock’s endurance is never truly inert, then; instead, it is structured according to what Cohen calls “animating antinomies”: “crystalline solidity and magmatic flow, geologic and idiosyncratic scale, blunt factuality and invitation to reverie” (p. 134). Although bedrock is often assumed to be the most unchanging, adamantine of substances, in its own time it is restless and forever in motion.

Still, any rush to claim a queer liveliness for the plasticity or even explosive qualities of bedrock, when viewed across geological timescales, would benefit from tarrying a little with its more inert qualities. For biological life can only subsist in relation to more deathly, inert states. Nigel Clark reminds us that only a small percentage of the earth’s rocky mantle – the crust – has cooled and solidified sufficiently to create “composite formations” (2018, p. 9) where life’s relation to the lithic might be experienced. Clark also notices a correlation between how bedrock hardens and cools so that the temporal and material fragility of the living might be preserved, with the evolution of homeostatic organisms that can exist as separate entities sustained in relation with an environment. As Clark puts it:

Just as the Earth composes a crust, a casing, around the unliveable energies of its interior, so too do all organisms establish a boundary between self and world – a skin, shell or husk that enfolds its metabolic system. Through this membrane passes the organic and inorganic matter through which the living body sustains itself, an exchange that must be controlled enough to hold the potentially overwhelming forces of the outer world at bay – but loose enough to admit the stimuli that can trigger transformation. (2018, p. 16)

Here, the geological and the lithic come surprisingly to resemble Freud’s own account of the death drive. For Freud proposes in *Beyond the Pleasure Principle* that the movement of the organism toward the inorganic from which it first primordially emerged is precisely what enables the organism to achieve homeostasis, as it shields itself from some of the fundamental excitation of life. The organism develops a carapace, perhaps we might even say a stony crust: “its outermost surface ceases to have the structure proper to living matter, becomes to some degree inorganic and thenceforward functions as a special envelope or membrane resistant to stimuli” (Freud, 1955, p. 27). Freud surmises that this carapace is fundamental to the origin of consciousness, which matches some of his earliest neurological accounts of a conservative tendency, or a principle of neuronal inertia, that produces and enables homeostasis (Freud, 1962a). Consciousness, or that which is figured as most complex, most alive in terms of the stratigraphic imaginary, can only emerge through an alliance with the inorganic that shields and enables another expression of life. Energy and incessant mutability must be tempered with the inertia of the inorganic – with death – for any life to find a way of existing in its own fashion.

## Opacity

The value of these insights on the work of the death drive is not, then, in articulating the bedrock psychic truth that “it is our nature to annihilate nature” (Kornbluh, 2024, para. 5). Instead, building on Elizabeth Povinelli’s (2016) account of the constitutive *geontological* separation of Life and Nonlife in late liberalism,^8^ we might come to see how the death drive interferes with an underlying framework that has placed a version of the living and sovereign human subject in an extractive relationship to the more-than-human world of the mineral. This dyad of Life (*bios*) and Nonlife (*geos*) is the propositional hinge of what Povinelli calls the “carbon imaginary,” which, in late liberalism, becomes “the fundamental ground of the governance of difference and markets” (Povinelli, p. 35). But would it be possible to think with relationships that are not always and already cauterized (to use Yusoff’s suggestive term) by the Life/Nonlife categories (Povinelli et al, 2017, p. 176)?

Placing her readings of Eurocentric philosophy alongside ideas drawn from working with Indigenous colleagues in the Belyuen community in Australia, Povinelli suggests other possibilities, not in the name of reshaping new ontological bedrocks, but as part of the work of sustaining other arrangements of existence and the demands embedded within them. In what she describes as a “dirty manifesto” for the analytics that have emerged from the Karrabing Indigenous social and aesthetic project, Povinelli foregrounds activities of endurance, persistence, and a permanent laboring over time to maintain relations that interfere with the stratifications of Life/Nonlife. As she writes: “Things exist through an effort of mutual attention. This effort is not in the mind but in the activity of endurance” (Povinelli, 2016, p. 28). The manifesto continues, insisting on preservation through perseverance over time that holds open other worlds:

We must dedramatize human life as we squarely take responsibility for what we are doing. This simultaneous de-dramatization and responsibilization may allow for opening new questions. Rather than Life and Nonlife, we will ask what formations we are keeping in existence or extinguishing? (Povinelli, p. 28)

As Povinelli points out elsewhere, the activity of endurance and the relations kept in existence or extinguished depend not on subjects or objects organised according to Life/Nonlife, but on the “creativity of keeping in place something that is constantly changing. Clearly this is a paradox. But my colleagues have always stressed this paradox. Nothing can ever be kept in place but can only be attended to” (Povinelli et al., 2017, p. 182). Attention must unfold over time.

So Povinelli is neither suggesting using animism to extend vitalism, for example, nor asking for the ethical obligations that currently attend Life and underpin liberal modes of recognition to be lent to the geological or the inorganic. This would be another way of negating Nonlife. Instead, she proposes interfering with the organizing principle of what currently underpins ethical and political understanding, action, and governance, by sustaining other arrangements and relations that decenter some versions of the human as the source of intention, sovereignty, rights, and extractive force. Povinelli stresses taking responsibility for sustaining relationships between life in death while “de-dramatizing” the tragic narrative of a sovereign subject, who is now becoming threatened by climate catastrophe and imagines itself as the synecdoche of imperilled planetary Life. Povinelli moves to assertively refusing late liberalism’s green initiatives, undertaken in relation to a point of crisis in historical time for liberal subjects and made in the name of a hopeful “progressive horizon” (2021, p. 46). The promises of these initiatives are almost always dependent on violence and loss for some so that others can continue to thrive. This is the bedrock of the bargain of liberal capitalism and colonialism, even though the “eternal returns” of that violence are represented as merely the “unintended, accidental, and unfortunate consequences of liberal democratic unfolding” (Povinelli, p. 41). Povinelli argues that even the philosophical and theoretical turns to states of relationality and entanglement in ecological thinking, which attempt to make violence harder to tolerate via radically expanded idea of liveliness, are in fact part of the “repetition compulsion of late liberalism, whereby the different toxic accumulations of racial and colonial catastrophes are refigured as a coming catastrophe for humanity that can be solved only by returning to a set of first conditions — to ontology” (p. 16). In other words, ontologies of multiplicity from the Global North tend not to foreground how colonialism and capitalism have already used, and continue to use, relationality, entanglement, and global connection to sequester the violence that underpins them, forcing multiple humans and more-than-human others to bear the losses that sustain certain configurations of Life only.

The turn to the Anthropocene, as an imaginary, holds the horror of extinction on the horizon. It thus remains a “geontological drama” that re-dramatizes the hierarchical distinctions that prioritize current designations of Life (Povinelli et al, 2017, p. 172). Suggestively, then, Povinelli’s final turn in *Geontologies* is to the radical capacities of Nonlife:

Life is not the miracle – the dynamic opposed to the inert of rocky substance. Nonlife is what holds, or should hold for us, the more radical potential. For Nonlife […] will fold its own extension back into the geological strata and rocky being, whereas Life can only fall into what already is. Life is merely a moment in the greater dynamic un-folding of Nonlife. And thus Life is devoured from a geological perspective under the pressure of the Anthropocene and Meteorocene. (Povinelli, 2016, p. 176)

Povinelli does not suggest an indifference to the destruction of the living. But she does insist on turning to non-capitalised forms of “life,” and to the temporalities of attention and maintenance, which might be bound up with inertia and lessness as much as with pleasure and unbound energy, to enable and sustain the preservation of other arrangements of existence.

Lisa Baraitser (2017; 2020) has argued that one way of figuring the time of attention, maintenance, and endurance required to preserve vulnerable forms of life would be via the suspension of the temporalities of action that underpin notions of liberal progress. These temporalities have contributed to climate crisis and ecocide, but they also shape the vital flurry of calls to “progressive” activity and “hope” mobilized by much climate activisim in the name of the future. Baraitser turns to the time of the “maternal” to track other forms of temporality and figure how practices of maintenance and care can emerge not simply from an alliance with a capitalized Life, but through forming a relationship with the inertia, suspensions, and repetitions of the death drive. Baraitser (2020) follows Freud and Johnston, noting that although repetition can be understood as deathly, as a way of negating life by turning away from development, it also contains elements of perseverance, preservation, and endurance that work through a tendency to shift and deviate, sustaining life rather than effecting a quick death. Baraitser uses the idea of the “maternal” (which here exceeds biological birthing) to supplement this account of the death drive by arguing that there also “needs to be the presence of another life that is prepared to wait whilst life and death come to have a relation to one another” (2020, p. 505): a “maternal” figure who attends to the “unfolding of another life in relation to one’s own path towards death” (p. 507). She suggests that the temporal practices of the maternal – practices of perseverance and maintenance bound to repetition that sustain life worlds – produce a permanent laboring and care that sustains and “animates ‘life’ in such a way as to allow the subject to die in its own fashion” (Baraitser, p. 503).^9^

Crucially, although this laboring includes a certain inertia – a suspension of one form of development and even a turning away from some kinds of immediate self-interest – the repetition that sustains something unfolding in its own fashion is a form of labor that is not a matter of indifference. As Baraitser writes, instead, “[m]aternity, in its failure to be indifferent to the specificity of its labour, implies a return, again and again, to a scene that matters” (2020, p. 509). What Baraitser outlines, through the relationality of the maternal, is a time of repetition that keeps on returning to the preservation and continuation of a relationship between life and death, or to “the time that time takes for one life to come to matter to another” (2017, p. 91). This creative “maternal” alliance with the death drive is not an ontological bid to see everything in relation; nor does it negate the diversity of human life in a gesture of indifference that would likely extend and intensify the biopolitical, necropolitical, and geontological violence and zones of sacrifice that underpin late liberalism. Turning to the death drive instead becomes a way of figuring how practices that endure over time, in and as a time of care, might require making alliances with inertia and repetition so that differences that matter can be allowed to unfold in their own time.

Although Baraitser describes this time of a maternal death drive as “crystalline” (2020, p. 505), in that it does not take part in the putative flow of developmental time, one might also want to think with the opacity of the “bedrock” of the death drive. Bedrock is usually figured as representing a limit for interpretation. One can see it but not through it. As I have argued elsewhere (Salisbury, 2011), Freud declined his position as a neurologist who depended on the hope for the transparency of some material structures (e.g, the cranium) but the opacity of others (e.g., the brain lesion or blood flow) to enable a neuroscientific empiricism based on seeing both form and function. He turned from the medical technological hope of being able to observe how mental functions can be mapped on to neurological matter toward diagnostic and clinical psychoanalytic listening that sought to interpret the dynamic qualities of the mind as they emerged, over time, from a mouth uttering language.

The death drive that the metapsychological Freud started to trace from 1920s onwards cannot be seen, mapped, and assimilated into the empirical gaze’s demand for transparency; it can only be interpreted through its detours and displacements. But there may be some value in that. The philosopher, poet, and critic Édouard Glissant (1997) used opacity as part of a theory of Relation that sought to preserve difference in the face of an urge for transparency and knowledge gained as clarity. As Glissant writes:

If we examine the process of “understanding” people and ideas from the perspective of Western thought, we discover that its basis is the requirement for transparency. In order to understand and thus accept you, I have to measure your solidity with the ideal scale providing me with grounds to make comparisons and, perhaps, judgements. I have to reduce. (Glissant, 1997, p. 189-190)

For Glissant, the Reason of Western thought repetitively invokes transparency in its compulsion to understand, categorize, and often stratify as part of its mode of acknowledgement and acceptance; it refuses to engage with difference in and of itself. But opacity disrupts the logic of transparency, supporting the constitutive importance of what Glissant calls an “unavailable world”. If opacity is allowed to inhere, weaves of Relation that coexist and converge might emerge, rather than simply an order of things. As he puts it: “one must focus on the texture of the weave and not on the nature of the components […] give up this old obsession with discovering what lies at the bottom of natures” (Glissant, p. 190).

Transparent logic and sites of colonialism, capitalism, and, indeed, liberalism have shaped the world, including the sense of the planet as a connected whole. Povinelli (2021) builds on Glissant’s work to argue that even ontologies of relationality and multiplicity that theory and philosophy have used to try to force a recognition of the sovereign subject’s entanglement with the planet,^10^ risk instituting yet another version of the transparency and boundlessness that have permitted otherness to be penetrated and violence to be sequestered in what are deemed to be zones of Nonlife. Nonlife might include so-called dead landscapes; the bedrock; or those humans who get to be imagined as a mineralogical resource, as hardly living at all (Yusoff, 2018). Glissant’s work shows, however, that a non-extractive engagement with Relation might begin with opacity and difference, and with historical and geographical specificities rather than ontologies. As Katherine McKittrick writes, there is a significant particularity in Glissant’s work that returns to sites of trauma, violence, and offence against Black existence in the Antilles: “He writes of the plantation, over and over. The plantation repeats. The precision of the plantation is everywhere” (2021, p. 7). These are sites and relations that matter in their difference and that need to be attended to over and again. Povinelli explicitly follows Glissant in asserting the importance of a fidelity to “actual worlds, regions of existence, and the actual ways these worlds and regions are entangled” (2021, p. 130), of returning to sites of ancestral catastrophe, to attend to ongoing powers to harm. She also insists on a fidelity to that which opaquely resists being swallowed up by geontological and necropolitical forces. Attending to repetitive practices of indigestible “stubbornness and survivance,” and incidences of fossilized opacity that resist analysis and interpretation through the Life/Nonlife dyad, might then be one way of following and maintaining relationships in and through time. Repeating, returning, and suspending progressive time in the name of the time it takes for forms and things to come to matter to one another, to use Baraitser’s suggestion of how an alliance might be formed with the death drive, might also open the possibility of other sets of relations. Under these conditions, what might get imagined as a mineralogical resource can come to matter and die in its own way, rather than be destroyed or swallowed up before its time.

## Endings

Toward the end of his life, Freud returned to the resistant opacity of the death drive as a bedrock, as a fundamental ground. But if we follow Glissant’s emphasis on particularity and power within opacity and Relation, we might avoid shaping the death drive into a concept that simply grounds a general ontology or ethics for the Anthropocene. Suggestive as turns to lithic liveliness are, for example, or Bersani and McGowan’s Lacanian emphases on a *jouissance* within the death drive that might be ethical because it breaches the boundaries of a sovereign subject’s self-interest, it is important to pay specific attention to how things come to matter over time and in the places, both biological and geological, that late liberal Life has sequestered as Nonlife – as a resource to be mined. As Povinelli puts it: “Nothing and no one may be a sealed subject, but simply repeating this fact makes no political headway” (2021, p. 33). Instead, in decentering and “de-dramatizing” formations of the human, and in attending to how Relation is woven and how things come to matter over time, there might be possibilities of holding back from forms of action that risk mortgaging the existence of many human and more-than-human others to the sustaining of a certain configuration of Life only. The repetitions of caring attention might work instead to sustain something so that it might die in its own fashion and in its own time, rather than be forced to die in the name of another version of Life.

Turning to psychoanalytic concepts can certainly be critiqued as a reassertion of anthropocentric modes of relating, but the death drive, as Freud noticed, forecloses the possibility of a utopian future for the human ratified under an idea of “civilization” and Life. The progressive horizons of action that undergird late liberalism, which are based on reaffirming, via geontological distinctions, what gets to matter, are also foreclosed by the death drive. But instead of understanding the death drive as simply an alibi for destructiveness, it might be used to ground other kinds of Relation – relations between a non-capitalized life and nonlife that go on alongside, sometimes inside, but also without “us.” Freud was certainly clear that the bedrock is not something that can be “mastered” in analytic treatment: “We can only console ourselves with the certainty that we have given the person analysed every possible encouragement to re-examine and alter his attitude to it” (1964, p. 253). Re-examining and altering one’s attitude, or we might say shifting and using one’s attention to see how things come to matter, seem like minimal, even disappointing gestures. These are neither ontologies nor technologies fit for the urgent timelines of saving a whole planet. Still, by using attention and care in a return to a scene that matters and the weaving of Relation, it may yet be possible to sustain something: to maintain for a time arrangements of existence that follow their own divergent and diverting paths back toward death.

## Footnotes

2 See, for example, Wynter & McKittrick (2015) and Yusoff (2018).

3 I use the term Anthropocene provisionally, recognizing the significant critiques made of the concept by Alaimo (2017) and others in Grusin (2017), and by Yusoff (2018).

4 Bersani did not elaborate on the “ecological” element in this formulation. But its position within his work on the self-shattering qualities of sexual *jouissance*, as “the organization of the self is momentarily disturbed by sensations or affective processes somehow ‘beyond’ those connected with psychic organization,” (Bersani, 1987, p. 217) suggests an ethics formed through the disintegration of sovereign subjectivity.

5 A bedrock implies a time frame that is not timeless, but one that nevertheless exceeds much human perception.

6 Malabou describes here a neurobiology that might be aligned with what Stacy Alaimo has influentially theorized as trans-corporeality, in which human embodiment is framed as a contact zone with the more-than-human environment. This corporeality is understood as embedded within and constructed from “the very stuff of the material, emergent world” (2010, p. 2, 20).

7 For Malafouris, there is a symmetry between the notion of embedment and concept of metaplasticity: “We create things that alter the ecology of our minds, re-configure the boundaries of our thinking and the ways we make sense of the world,” as part of the “ongoing relational transaction at the heart of human becoming” (2015, p. 351).

8 Povinelli uses “late liberalism” to describe the contemporary phase of neoliberal governance, ethics, and politics. “Late liberalism” is grounded not simply on the Foucauldian biopolitical regulation of human populations, but on a fundamental ontological distinction between what is figured and valued as living, and what is not.

9 For a contrasting reading of the ecofeminist potential of the maternal that emphasises connectivity, entangled materialisations, and embodied relatedness, see Phillips (2017).

10 See, for example, Barad (2007).
